# Guiding Hypertension Management Using Different Blood Pressure Monitoring Strategies (GYMNs study): comparison of three different blood pressure measurement methods: study protocol for a randomized controlled trial

**DOI:** 10.1186/s13063-019-3366-8

**Published:** 2019-05-10

**Authors:** Hao-Min Cheng, Shih-Hsien Sung, Chen-Huan Chen, Wen-Chung Yu, Shu-Mei Yang, Chao-Yu Guo, Shao-Yuan Chuang, Chern-En Chiang

**Affiliations:** 1 Center for Evidence-based Medicine, Taipei Veterans General Hospital, No. 201, Sec. 2, Shih-Pai Road, Beitou District, Taipei, 112 Taiwan, Republic of China; 2 Divison of Faculty Development, Taipei Veterans General Hospital, Taipei, Taiwan; 3 Department of Medicine, Taipei Veterans General Hospital, Taipei, Taiwan; 4 Department of Medicine, National Yang-Ming University, Taipei, Taiwan; 5 Institute of Public Health, National Yang-Ming University, Taipei, Taiwan; 6 Division of Preventive Medicine and Health Service, Research Institute of Population, Health Sciences, National Health Research Institutes, Miaoli, Taiwan; 7 General Clinical Research Center, Taipei Veterans General Hospital, No. 201, Sec. 2, Shih-Pai Road, Beitou District, Taipei, 112 Taiwan, Republic of China

**Keywords:** Unattended automated office blood pressure, Central blood pressure, Home blood pressure, Sphygmomanometer, Blood pressure monitoring

## Abstract

**Background:**

Home blood pressure (BP) and unattended automated BP (uAOBP) monitoring have been recommended by guidelines for the care of hypertensive subjects. However, BP measurements in the peripheral arteries cannot serve as direct substitutes for their central counterparts. Moreover, the comparative effectiveness and safety of BP-guided strategies using these BP measuring devices have never been evaluated.

**Methods/design:**

Patients with uncontrolled or newly diagnosed hypertension aged 20–90 years will be recruited via outpatient clinics and allocated into three arms by stratified randomization (baseline systolic BP 130–155 mmHg and 155–180 mmHg): home BP, uAOBP, and central BP-guided treatment. At each scheduled visit to the clinic, a patient’s BP will be measured by each of the three methods of measuring BP. The blood pressure from three different methods will be confirmed available at each visit. Patients and physicians will be blinded to the allocated interventions because they will use measured BP values in the clinic through a standardized report format. A common BP target for systolic blood pressure (SBP) of 130 mmHg is adopted for these BP-guided strategies. The primary outcome is the change of 24-h mean ambulatory SBP at 3 months. A key secondary outcome is to determine the percentage achieving their target BPs at 3 months and the decrease of left ventricular mass at 12 months.

**Discussion:**

To our knowledge, this is the first prospective double-blind randomized controlled trial to assess the optimal guiding strategy for hypertension. It will help to define which BP monitoring method is the most effective for guiding the clinical management of hypertension. It will provide good evidence to support future guideline recommendations for BP monitoring devices.

**Trial registration:**

ClinicalTrials.gov, NCT03578848. Registered on 4 June 2018.

**Electronic supplementary material:**

The online version of this article (10.1186/s13063-019-3366-8) contains supplementary material, which is available to authorized users.

## Background

Throughout middle and old age, blood pressure (BP) is strongly and directly related to vascular and all-cause mortality [1]. Lowering high BP has been shown to significantly reduce the risk of cardiovascular disease [2]. However, the traditional way of measuring BP in clinical practice, the office BP, is usually done in a busy and hurried clinical environment, and is susceptible to the well-known confounding whitecoat effect [3, 4]. As such, unattended automated office BP (uAOBP) monitoring has been proposed as an effective way to measure BP [4] and further, has been promoted by Canadian physicians [5]. Nonetheless, out-of-office BP, home BP, and ambulatory BP monitoring remain the recommended methods for mitigating the whitecoat effect [6–8], and their prognostic value has been demonstrated to be superior to the traditional office BP [9]. In a previous systematic review and meta-analysis, home BP has been shown to be as good as ambulatory BP in predicting target organ damage [10] and a better guiding strategy than conventional office BP [11]. Home BP monitoring, with its ability to detect morning and masked hypertension, has a better tolerability than ambulatory BP monitoring for long-term use. It could, therefore, be a strategy of choice and replace office BP monitoring for guiding hypertension management.

Moreover, BP measurements in the peripheral arteries cannot serve as direct substitutes for their central counterparts because of the long-recognized differences in blood pressure waveforms [12] and values [13] between the central aorta and the peripheral arterial system. Thus, if decisions to adjust medication are made solely based on brachial BP, there could be a considerable risk of over- or undertreatment [14].

Considering that there are many better strategies for guiding hypertension management than traditional office BP, there is an apparent need to investigate their comparative effectiveness and safety in the management of hypertension. We hypothesize that home BP may be non-inferior to uAOBP and central BP-guided in reducing ambulatory BP, which the present randomized controlled trial is designed to test.

## Methods/design

### Study design and rationale for the reference standard: invasively measured central BP

This is a 12-month prospective double-blind parallel-group randomized trial. The study is scheduled to commence in June 2018. We plan to enroll a total of 252 patients with 84 subjects allocated to each of the three arms. The uAOBP and CBP groups will be compared to the Home BP group for data analysis. Details of the sample size calculation is provided in the statistics section (Section 2.6).

### Study population

Patients with hypertension will be recruited at outpatient clinics, from advertisements, and at Taipei Veterans General Hospital. The inclusion criteria are as follows:

- 20 to 90 years of age
- not pregnant
- receiving antihypertensive therapy for uncomplicated essential hypertension and taking one or two types of antihypertensive drugs (to rule out complicated or resistant hypertension) or with hypertension newly diagnosed by uAOBP (uAOBP > 130 mmHg at the screening visit)

The exclusion criteria are as follows:

- poor adherence to medication
- unable to conduct self-measurement of blood pressure
- history of polycystic kidney disease
- congestive heart failure (a recent assessment of left ventricular (LV) ejection fraction <40% prior to the screening visit)
- chronic kidney disease with estimated glomerular filtration rate <30 mL min^-1^ 1.73 m^-2^ (using the method from the Modification of Diet in Renal Disease study) at the screening visit
- recently recorded severely abnormal LV mass index (>59 g/m^2.7^ in women and >64 g/m^2.7^ in men) prior to the screening visit
- secondary causes of hypertension
- uncontrolled hypertension (uAOBP > 180/100 mmHg at the screening visit)
- history of severe aortic valve disease
- history of upper limb obstructive atherosclerosis
- history of atrial fibrillation
- BP differences more than 5 mmHg between both arms at the screening visit

Patients will be asked to sign the informed consent by the designated investigator before the interview. Eligible participants will be paid US $16.5 in cash at the baseline visit and at the end of study visit. Routine blood and urine tests will be analyzed at baseline and at the end of the study.

### Study protocol

Patients will be randomized to have hypertension management decisions made based on uAOBP, home BP, or central BP monitoring. Permuted block randomization will be done using computer-generated randomization codes in two stratums with baseline systolic BP (uAOBP) 130–155 mmHg and 155–180 mmHg. Whether the BP measurements are made on the left or right arm will also be determined by randomization codes. The BP for each patient will be measured by the three different methods of measurement at each visit. To guide the management of hypertensive patients, adjustments to medication will be based on a published guideline [8] using the BP measured by the different monitoring methods. The use of different devices to obtain the BP for guiding the care of hypertensive patients aims to determine whether these different methods are of comparable clinical value in routine practice. To achieve this, intervention patients will have their medication titrated to normalize these different BP values. The target BP level, systolic blood pressure (SBP) of 130 mmHg, of these different BP monitoring strategies is based on the latest guideline [8, 15, 16].

**Fig. 1:**
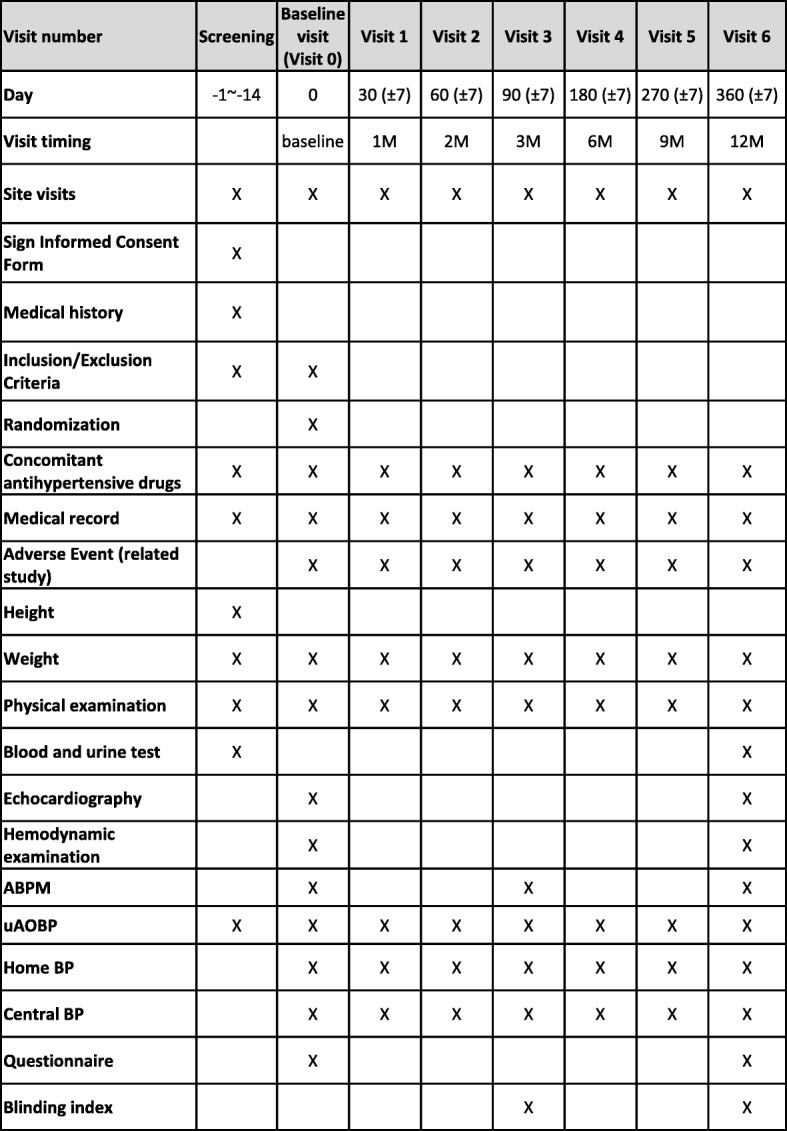
Schedule of assessment. ABPM ambulatory blood pressure monitoring, BP blood pressure, M month, uAOBP unattended automated BP

An overview of the study protocol comply with the Standard Protocol Items: Recommendations for Interventional Trials (SPIRIT) guidelines (Additional file 1) and acquired measures is presented in Fig. 1. Although we encourage physicians to adjustment medication following the practice guideline [8, 16], the decision will be left to the discretion of the patient’s attending doctor. During the periods when BP is monitored at home, no adjustment to the medication is allowed to preserve the comparability between different BP values. At each scheduled visit, the corresponding BP will be measured before the patient meets their doctor and it will be used to adjust the patient’s medication according to the practice guideline [8, 16].

In summary, if the measured SBP is well below the target BP and there are no side effects possibly caused by the antihypertensive agents, no adjustment to medication is required. If the measured SBP is higher than the target BP by less than 20 mmHg, the dose of one drug only will be increased by the suggested maximal dose amount. If the measured SBP is higher than the target BP by between 20 and 40 mmHg, then the doses of two drugs (or a single-pill combination) will be increased and if the difference is above 40 mmHg, the doses or three drugs (or a single-pill combination) will be increased. If the BP is low (less than 90/70 mmHg) or there are possible drug-related side effects, a cautious adjustment of the prescribed antihypertensive will be made.

The patients are asked to bring their medication to each visit so that their pills can be counted. All dose adjustments and side effects will be recorded throughout the whole study period. A sub-study will test the level of agreement between the different methods of measuring BP.

### BP monitoring: uAOBP, central BP, and home BP

At the initial visit, the patient’s BP will be measured simultaneously from both upper arms by an oscillometric BP monitor (WatchBP Office Central; Microlife AG, Widnau, Switzerland). Subjects with SBP differences between both arms of more than 5 mmHg will be excluded. The procedure for BP measurements will adhere to the standard procedure [6].

For uAOBP and central BP, the measurements will be conducted in a quiet room without the presence of clinical personnel. The patients will be seated without talking. The BP used will be the average of three measurements taken with an automated device (HEM-907, Omron Healthcare, Lake Forest, IL) that has been preset to wait 5 min between measurements [3]. Simultaneously, the central BP will be measured in the other upper arm (WatchBP Office Central; Microlife AG, Widnau, Switzerland) [17]. Whether to use the right or left upper arm for uAOBP or central BP will be based on computer-generated random codes before enrollment.

For the home BP measurements, a validated device (WatchBP Home; Microlife AG, Widnau, Switzerland) will be given to all subjects allocated to measure BP at home BP. Subjects are requested to take their BP in the morning (within 2 h after awakening) and evening (between 6 pm and midnight), before taking their medication, for 7 consecutive days before each scheduled clinical visit. The assistant will check the record of home sphygmomanometer at each visit. The BP taken on the first day of the measurements will be discarded and an average of all BP readings taken in the morning and in the afternoon will be calculated and given to the clinicians to guide treatment.

The ambulatory BP of all subjects will be measured at baseline, at 3 months, and at the end of the study by a validated device (WatchBP O3 AFIB Ambulatory). Similarly, the choice of right or left arm for the measurement of ambulatory BP will be determined randomly. All the automated BP readings will be stored digitally for analysis and the BP values for each arm will be given to clinicians to guide hypertension management.

### Randomization, outcomes, and masking

Each patient will be randomly assigned, using a standard computer protocol at the General Clinical Research Center, Taipei Veterans General Hospital, Taipei, Taiwan, to the interventions in a 1:1:1 ratio using sealed opaque envelopes (sequentially numbered). The study coordinator will oversee the enrollment and intervention assignment and maintain the concealment of the allocation.

The primary outcome is the change in 24-h mean ambulatory SBP at 3 months. The secondary outcomes include: (1) the change in 24-h mean ambulatory diastolic blood pressure (DBP) at 3 months, (2) decrease in LV mass at 12 months, (3) change in SBP/DBP measured by uAOBP monitoring, home BP monitoring, or central BP monitoring, (4) change in quality of life, (5) changes in medication, and (6) side effects. Any possible treatment-related side effects, including hypotension, injurious falls, dizziness, electrolyte imbalances (serum Na < 130 meq/L and serum K > 5.5 or < 3.0 meq/L), syncope, acute renal failure (increase in serum creatinine > 1.5 times baseline, or increase in serum creatinine by ≥0.3 mg/dL), and bradycardia (heart rate <50 bpm detected by electrocardiogram or ECG), will be recorded and evaluated. The quantity of medication taken will be recorded using the daily defined dose (DDD) calculated as per the World Health Organization standard. The DDD is a statistical measure of drug consumption used to standardize comparisons across drug classes, e.g., 1 × DDD = 150 mg irbesartan or 5 mg amlodipine [18].

Patients will be interviewed by well-trained interviewers using a structured questionnaire. Questions are on sociodemographic characteristics, smoking habits, consumption of alcohol, tea, and coffee, vegetarian habits, sleep, physical activity, medical history, and medication history. The quality of life will be assessed by the Taiwan version of the Short Form 36 (SF-36) [19]. The Taiwan version of the International Physical Activity Questionnaire Short Form (IPAQ-SF) [20, 21] will be used to examine physical activity in patients. The nutritional status of elderly patients will be assessed using the Mini Nutritional Assessment [22]. These questionnaires will be used at baseline and at 12 months. The effectiveness of blinding will be assessed using the blinding index [22] at 3 months and at 12 months. A two-dimensional echocardiograph will be taken with an Artida Echocardiograph (Toshiba Medical Systems Corporation, Tokyo, Japan) and end-diastolic LV dimensions will be used to calculate LV mass by an anatomically validated formula [23], and subsequently normalized body height^2.7^ [24].

The attending physicians will remain blinded to the allocation because they will be given the measured BP through a standardized report form and will not know which BP monitoring device was used. Except for patient-reported outcomes and adverse events, the investigators and participants are blinded to all outcome variables (which will be calculated at the end of the study). The assessment of LV mass will be conducted on side-by-side images by a technician blinded to allocation.

### Data analysis and statistical analyses

Based on data from our previous work [25], the sample size (84 participants per group) was determined on the basis of non-inferiority between three independent groups (α = 0.05; β = 0.20; standard deviation of ambulatory BP 11 mmHg; mean difference and non-inferior margin 5 mmHg, drop rate 10%).

A clinical study information system is used to manage this clinical trial. Access to the data system is restricted to members of the research team only. We will test the normality of all parameters using the Shapiro–Wilk test. Categorical data will be presented as proportions, and continuous data as means and standard deviations or as medium and interquartile ranges when appropriate. Data for all randomized patients in the three arms will be analyzed based on the intention-to-treat principle. For continuous variables (LV mass index, 24-h ambulatory BP, heart rate, and quality of life), the analysis will be undertaken using linear regression, with the dependent variable calculated as the change over time. We will analyze DDD medication data recorded at all visits using mixed regression models to account for repeated measures on individuals over time, with outcome variables log-transformed to correct for heteroskedasticity where necessary. We will present back-transformed means and confidence intervals. Recommendations for medication use at each visit were categorized as maintain, increase, decrease, or cease [14]. A log-multinomial regression model will be used to assess the group differences for each of the three arms, with clustering by individual to account for repeated measures over time. A χ^2^ test will be used to determine the relation between categorical variables. Intra-class correlations and a paired *t*-test will be used to assess the agreement between different strategies.

### Data safety monitoring

We have established a data and safety monitoring board to monitor all aspects of the study. All issues related to participant safety will be reported by the medical safety officer to the independent data and safety monitoring board, which will monitor data and oversee participant safety. The board will meet twice a year to monitor safety. It will advise the research steering committee on study progress and performance, and make recommendations regarding whether the study should continue or if there should be a protocol change. The data and safety monitoring board will attempt to identify any major adverse outcomes due to the therapy. The following are possible safety events relating to the intervention:

- serum sodium ≤132 or >150 meq/L
- serum potassium <3.0 or >5.5 meq/L
- serum creatinine increased by at least 50% to ≥1.5 mg/dL since the last blood sample test
- heart rate <40 bpm
- ECG shows complete heart block or bradycardia <40 beats/min
- injurious falls
- syncope
- any unexpected event that the investigator believes could be attributed to the intervention

Serious adverse events are any adverse events that meet any of the following criteria:

- fatal or life-threatening
- resulting in significant or persistent disability
- requiring or prolonging hospitalization
- any events that investigators judge are significant hazards or harm to the participants

### Ethics and dissemination

The GYMNs trial was approved by the institutional review board at Taipei Veterans General Hospital (2018–05-009A) on 29 May 2018. The findings of this study will be published in peer-reviewed journals. It will provide good evidence to inform future guideline recommendations for BP monitoring devices.

## Discussion

### Rationale

Taking BP measurements is a clinical procedure of considerable importance because it serves as an imperative foundation in the management of hypertension, which is the most significant cardiovascular risk factor across the globe [26]. However, a substantial whitecoat effect, which is the difference between office and out-of-office BP, can be observed when measuring in BP in routine clinical practice. This can make correctly adjusting the prescription of antihypertensive agents a challenging task [27]. Home BP and uAOBP monitoring have been confirmed as successful methods that eliminate the whitecoat effect. In addition, the central BP has been shown to be better than the conventional office BP in predicting cardiovascular risk [28] and it may be a more cost-effective method for diagnosing hypertension [29]. In the era of evidence-based medicine, clinical trials are required to investigate the comparative effectiveness between these readily available BP monitoring strategies to inform clinical practice decisions [30]. Using the gold standard of BP monitoring, the ambulatory BP, as the primary endpoint [31], we suggest home BP monitoring may be non-inferior to uAOBP and central BP monitoring as a guiding tool in the management of hypertension. Home BP is obtained by consecutive measurements and therefore, is associated with a better accuracy and prognostic value than conventional office BP. Moreover, home BP monitoring is easier to do than uAOBP. If a comparable effectiveness could be demonstrated, it may have the potential to become the standard guiding procedure for hypertension.

### Challenges in using office BP to guide the clinical management of hypertension

Using conventional office BP in the management of hypertension is heavily affected by the busy and hurried clinical environment. In a previous review article, it was demonstrated that routine office BP is substantially higher than research office BP, uAOBP, and daytime ambulatory BP [32]. Therefore, it could be risky and imprudent to titrate antihypertensive agents based solely on routine office BP. As such, many alternative strategies have been proposed to replace conventional office BP to guide the management of hypertension [11, 33–35]. Due to its feasibility and effectiveness, home BP might be the strategy of choice in the care of hypertensive subjects. However, its comparative effectiveness and safety in comparison with uAOBP and central BP monitoring have never been evaluated.

### Is uAOBP the best BP measurement technique?

uAOBP, with its potential to eliminate the whitecoat effect, has been adopted in the SPRINT study [3, 36]. One may partly attribute the success of the SPRINT study to the use of this more accurate BP measurement technique. It is, therefore, a promising technique for routine clinical practice. However, in some clinical settings, it is probably unrealistic to implement uAOBP given its requirement for time and space, and device costs. Without a randomized control trial comparing the effectiveness and safety of different BP monitoring strategies, it is difficult to make an evidence-based decision to guide the clinical management of hypertension.

### Double-blind versus open-label design for the treatment strategies

We designed this study as a double-blind study. Clinical information will be provided to clinicians without them knowing which method was used to measure their patients’ BP. The allocation concealment and blinding of patients, caregivers, and outcome accessors will be rigorously kept to avoid any possible placebo or performance bias. To our knowledge, this may be the first double-blind randomized controlled trial to evaluate the best BP monitoring strategy.

### Blood pressure threshold

We adopt a common BP target based on the recommendation of the latest hypertension guideline for uAOBP and home BP [6]. Usually, central BP is lower than brachial BP. However, there are two types of central BP device according to whether BP amplification is preserved: some devices give an estimate of central BP relative to measured brachial BP (type I) while others estimate the intra-arterial central BP (type II) [37]. We previously conducted a survey on the prevalence of hypertension, the 2013–2016 National Nutrition and Health Survey in Taiwan [38]. In this national representative cohort, a type 2 central BP device was used to measure central BP. As shown in this study, central and brachial SBP/DBP had similar values. We, therefore, decided to use the same BP target for central BP monitoring to guide hypertension treatment.

### Limitations

Although we will count the pills held by a patient at each visit, poor medication adherence patients may be a limitation of this trial.

### Conclusion

The GYMNs trial is ongoing and due to complete in 2021. The trial should be fully powered to test its primary hypothesis. It is the first double-blind randomized controlled trial to evaluate the optimal guiding strategy for hypertension and it will help define which method of BP monitoring is the most effective in guiding the clinical management of hypertension. Whatever the outcome, the findings of GYMNs are likely to influence future international guidelines for the choice of BP monitoring strategy in routine clinical practice in the care of hypertensive subjects.

### Trial status

This protocol is version 1, dated 19 March 2018. Recruitment began on 6 June 2018. We planned to achieve the recruitment target by December 2021.

## Additional file

Additional file 1: SPIRIT 2013 Checklist: Recommended items to address in a clinical trial protocol and related documents. (DOC 120 kb)

## Abbreviations

BP: Blood pressure
DBP: Diastolic blood pressure
DDD: Daily defined dose
ECG: Electrocardiogram
IPAQ-SF: International Physical Activity Questionnaire Short Form
LV: Left ventricular
SBP: Systolic blood pressure
SF-36: Short Form-36
uAOBP: Unattended automated office blood pressure
